# Antioxidant, Antiproliferative and Apoptosis-Inducing Efficacy of Fractions from *Cassia fistula* L. Leaves

**DOI:** 10.3390/antiox9020173

**Published:** 2020-02-20

**Authors:** Sandeep Kaur, Ajay Kumar, Sharad Thakur, Kapil Kumar, Ritika Sharma, Anket Sharma, Prabhpreet Singh, Upendra Sharma, Subodh Kumar, Marco Landi, Marián Brestič, Satwinderjeet Kaur

**Affiliations:** 1 Department of Botanical and Environmental Sciences, Guru Nanak Dev University, Amritsar 143005, India; soniasandeep4@gmail.com (S.K.); kumar.ajay1250@gmail.com (A.K.); anketbot.rsh@gndu.ac.in (A.S.); 2 Department of Molecular Biology and Biochemistry, Guru Nanak Dev University, Amritsar 143005, India; thakursharad23@gmail.com; 3 Department of Chemistry, Guru Nanak Dev University, Amritsar 143005, India; kapil.chem88@gmail.com (K.K.); prabhpreet1979@gmail.com (P.S.); subodh_gndu@yahoo.co.in (S.K.); 4 School of Pharmaceutical Sciences, Lovely Professional University, Punjab 144411, India; 5 Natural Product Chemistry and Process Development division, CSIR-IHBT, Palampur 176061, India; ritikas556@gmail.com (R.S.); upendraihbt@gmail.com (U.S.); 6 State Key Laboratory of Subtropical Silviculture, Zhejiang A&F University, Hangzhou 311300, China; 7 Department of Agriculture, Food and Environment, University of Pisa, 56124 Pisa, Italy; 8 Interdepartmental Research Center Nutrafood “Nutraceuticals and Food for Health”, University of Pisa, 56124 Pisa, Italy; 9 CIRSEC, Centre for Climatic Change Impact, University of Pisa, 56124 Pisa, Italy; 10 Department of Plant Physiology, Faculty of Agrobiology and Food Resources, Slovak University of Agriculture, 94976 Nitra, Slovakia; marian.brestic@uniag.sk; 11 Department of Botany and Plant Physiology, Faculty of Agrobiology, Food and Natural Resources, Czech University of Life Sciences, 16500 Prague, Czech Republic

**Keywords:** *Cassia fistula*, antioxidant, antiproliferative, apoptosis, osteosarcoma

## Abstract

Cassia fistula L. is a highly admirable traditional medicinal plant used for the treatment of various diseases and disorders. The present study was performed to divulge the antioxidant, antiproliferative, and apoptosis-inducing efficacy of fractions from *C. fistula* leaves. The hexane (CaLH fraction), chloroform (CaLC fraction), ethyl acetate (CaLE fraction), *n*-butanol (CaLB fraction), and aqueous (CaLA fraction) were sequentially fractionated from 80% methanolic (CaLM extract) of *C. fistula* leaves. The CaLE fraction was fractionated using column chromatography to yield a pure compound, which was characterized as Epiafzelechin (CFL1) based on ^1^H, ^13^C, and DEPT135 NMR. Among these fractions, CaLE and isolated CFL1 fractions exhibited an effective antioxidant potential in Ferric ion reducing power, (2,2’-azino-bis (3-ethylbenzothiazoline -6-sulfonic acid)) cation radical scavenging, and nitric oxide radical scavenging assays. Epiafzelechin was investigated for its antiproliferative effects against MG-63 (osteosarcoma), IMR-32 (neuroblastoma), and PC-3 (prostate adenocarcinoma), and was found to inhibit cell proliferation with a GI_50_ value of 8.73, 9.15, and 11.8 μM respectively. MG-63 cells underwent apoptotic cell death on treatment with Epiafzelechin as the cells showed the formation of apoptotic bodies, enhanced reactive oxygen species (ROS) generation, mitochondrial membrane depolarization along with an increase in early apoptotic cell population analyzed using Annexin V-FITC/PI double staining assay. Cells showed cell cycle arrest at the G_0_/G_1_ phase accompanied by a downregulation in the expression levels of p-Akt (Protein kinase B), p-GSK-3β (Glycogen synthase kinase-3 beta), and Bcl-xl (B-cell lymphoma-extra large) proteins. RT-PCR (Real time-polymerase chain reaction) analysis revealed downregulation in the gene expression level of β-catenin and CDK2 (cyclin-dependent kinases-2) while it upregulated the expression level of caspase-8 and p53 genes in MG-63 cells.

## 1. Introduction

Flavonoids, secondary plant metabolites with antioxidant properties have been found to possess multiple molecular mechanisms of action by modulating different receptors and enzymes in signal transduction pathways related to cell proliferation, inflammation, differentiation, angiogenesis, metastasis, apoptosis, and reversal of multidrug resistance [1]. Cancer is considered a multifactorial disease that is normally characterized by the deregulation of cellular signaling pathways, uncontrolled growth, and the spread of malignantly transformed neoplastic cells [2]. Interestingly, certain experimental and practical approaches of traditional medicinal plants have identified that the secondary metabolites present in the host plant act as a natural defense system and possess antioxidant, anti-inflammatory, antiproliferative, and chemopreventive properties [3]. In several instances, phytochemicals have been observed to show pro-oxidant properties concerning cancer treatment [4]. Mounting evidence has supported that phytochemicals interfere with the signaling pathways, and finally regulate the downstream proteins/genes associated with specific cell behavior: for instance, CDK (cyclin-dependent kinases) and cyclins are related to cell proliferation, Bcl-2 family proteins, Bax and caspases are related to apoptosis, Myc (proto-oncogene) is related to cell cycle arrest, and MMPs (matrix metalloproteinase) are related to invasion [5,6]. The participators along the signaling pathways can be regulated via expression (at epigenetic, genetic, or translational levels) and activation (cleavage and phosphorylation). 

Osteosarcoma (OS) is considered the third most common cancer, which mostly affects children and young adults [7,8]. The annual incidence of osteosarcoma is 5.6 million cases per child [9], in which it increases to 8–11 per million per year at 15–19 years of age [10]. OS normally arises due to uncontrolled cell proliferation, defects in cell cycle regulation, mutations in DNA helicases and tumor-suppressor genes, and about 70% specimens of the tumor revealed chromosomal abnormalities [9,11]. 

*Cassia fistula* L. is a medicinal plant of the family Fabaceae commonly known as Amaltas; the Golden Shower tree has been extensively used in the traditional medicinal system for treatment of skin diseases, rheumatism, liver troubles, malaria, jaundice, anorexia and other inflammatory diseases [12]. Epiafzelechin, a flavan-3-ol, was isolated from *Cassia fistula* L. from the CaLE fraction harboring antioxidant-rich phytoconstituents. The present study was planned to unravel the potential of Epiafzelechin for its antiproliferative and apoptosis-inducing activity in Human osteosarcoma (MG-63) cells. This is the first report of the antiproliferative and apoptosis-inducing effects of Epiafzelechin in Human osteosarcoma cells.

## 2. Materials and Methods

### 2.1. Chemicals and Reagents 

Dulbecco’s modified Eagle’s medium (DMEM), paraformaldehyde, hexamethyldisilazane, Hoechst 33342, propidium iodide (PI), glutaraldehyde, Fluoromount, 2′,7′-Dichlorodihydrofluorescein diacetate (DCFH-DA), and Rhodamine-123 were obtained from Sigma (St. Louis, MO, USA). 3-(4,5-dimethylthiazol-2-yl)-2,5-diphenyl tetrazolium bromide (MTT) and trypsin were obtained from himedia Pvt. Limited (Mumbai, India). Fetal Bovine Serum (FBS) was purchased from Biological industries, Cromwell, CT, USA. Rabbit monoclonal Bcl-xl, p-Akt, p-GSK-3β antibodies, and anti-rabbit HRP (Horseradish Peroxidase)-labeled secondary antibody were purchased from Cell Signaling Technology, Danvers, MA, USA. Primers, SYBR and cdna synthesis kit were purchased from Bio-rad, California, USA. The BD Cycletest plus DNA Kit (BD Biosciences) and fluorescein isothiocyanate (FITC)-conjugated Annexin V/PI assay (BD Pharmingen Annexin V FITC apoptosis detection kit) were obtained from BD Biosciences, San Jose, CA, USA. All reagents used to perform the experiments were of analytical (AR) grade.

### 2.2. Collection and Authentication

The *C. Fistula* leaves were collected in the month of May from the Guru Nanak Dev University, Amritsar, India. The authentication of the plant leaves and their botanical identification were made in the Herbarium of the Department of Botanical and Environmental Sciences, Guru Nanak Dev University, Amritsar, and voucher specimens with accession No. 6782 have been deposited in the Herbarium.

### 2.3. Extraction/Fractionation of C. Fistula Leaves 

**Scheme 1:**
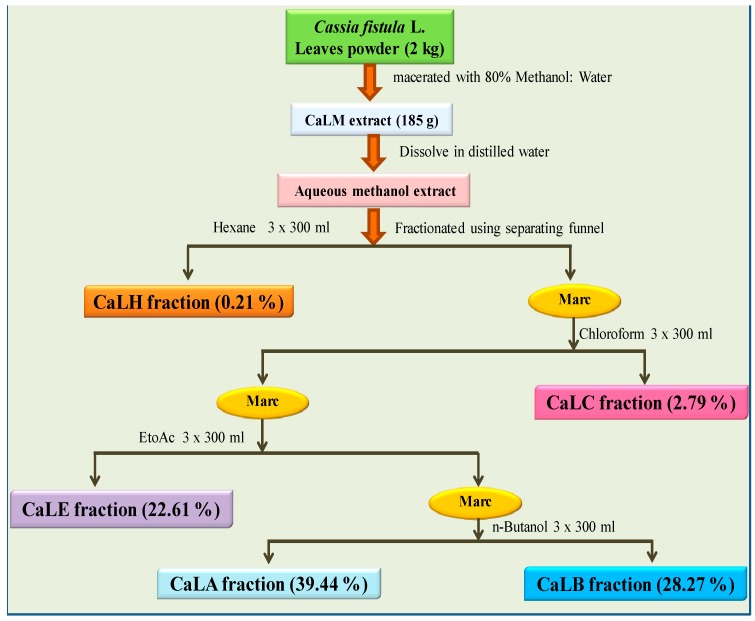
Isolation of extract/fractions from them leaves of *Cassia fistula* L.

The leaves were thoroughly washed under tap water, followed by drying at room temperature and crushed to a coarse powder. The *C. fistula* leaves powder (2 kg) were extracted by employing the maceration method using 80% methanol and then filtered with the help of the Whatman no. 1 filter paper. The solvent of the aqueous methanol extract was evaporated under reduced pressure by using a Rota-vapor (Buchi R-210, Flawil, Switzerland) to obtain an aqueous methanolic extract named the CaLM extract (95 g). The obtained dried extract was dissolved in double-distilled water and further fractionated in separating funnel. The fractionation was carried out in the increasing order of polarity viz. *N*-hexane, chloroform, ethyl acetate, and *n*-butanol to yield the CaLH fraction (0.21%); CaLC fraction (2.79%); CaLE fraction (22.61%); CaLB fraction (28.27%), and remaining CaLA fraction (39.44%) respectively (Scheme 1). 

### 2.4. Antioxidant Assay

#### 2.4.1. Ferric Ion Reducing Antioxidant Power (FRAP) Assay

The reducing potential of the fractions isolated from *Cassia fistula* L. was performed according to the method described by Oyaizu [13]. In this assay, different concentrations (50–800 μg/mL) of the test sample were taken in the test tube in triplicates. To this, 0.2 M of phosphate buffer was added (1 mL, pH 6.6) and 1% of Potassium ferricyanide solution (1 mL). The reaction mixture was mixed thoroughly and incubated for 15–25 min at 50 °C. After incubation, added 10% trichloroacetic acid (1 mL) followed by centrifugation for 10 min at 4500 rpm. The supernatant obtained was collected, and to this, 3 mL of double distilled water was added followed by the addition of 0.1% ferric chloride (0.5 mL). Finally, the absorbance was read at 700 nm with the help of the Ultraviolet-Visible spectrophotometer (Systronics 2202 UV–Vis, Gujarat, India). The increase in the reducing ability of the sample was due to an increase in the absorbance of the reaction mixture, and the results obtained were compared with standard antioxidant ascorbic acid.

#### 2.4.2. ABTS (2,2’-azino-bis(3-ethylbenzothiazoline-6-sulfonic acid)) Cation Radical Scavenging Assay

The assay is based on the reduction of the green ABTS•^+^ to colorless ABTS [14]. The ABTS stock solution (7 mM) was added to 2.45 mM potassium persulfate to generate ABTS cation by allowing the mixture to be oxidized for 15–17 h at room temperature in the dark before use. The oxidized ABTS cation solution was diluted with ethanol to an absorbance of 0.70 (± 0.02) at 734 nm. After this, 300 μL of the fraction was added to 1.0 mL of the ABTS cation solution. The decrease in the absorbance was read after 5 min using a spectrophotometer (Systronics 2202 UV–Vis spectrophotometer, Gujarat, India). The ABTS cation solution was taken as a blank. The standard antioxidant used was L-ascorbic acid. The antiradical activity of fractions of *C. fistula* was expressed as percentage inhibition of ABTS•^+^, which was calculated according to the formula mentioned below.

Percentage inhibition = (A_0_ − A_1_)/A_0_ × 100

where A_0_ is the absorbance of ABTS•^+^ solution + vehicle solvent (control); A_1_ is the absorbance of the reaction mixture (containing the different concentrations of a fraction and ABTS•^+^ solution).

#### 2.4.3. Nitric Oxide Radical Scavenging Assay

The nitric oxide radical scavenging efficacy of *C. fistula* fractions was determined according to the sodium nitroprusside method of Garrat et al. [15] with slight modifications. The reaction mixture contained 5 mM sodium nitroprusside prepared in PBS, pH 7.4 (0.3 mL) and mixed with different fraction concentrations (50–800 µg/mL) followed by 30 min of incubation at 37 °C. After completion of incubation, 2 mL of Griess reagent (2% H_3_PO_4_ and 1% sulfanilamide) was added and again incubated for 20 min at room temperature. Then, 50 µL of N-(1-naphthyl) ethylenediamine dihydrochloride (0.1%) was added and the absorbance of the chromophore was measured at 546 nm. The standard antioxidant used was L-ascorbic acid. The nitric oxide radical scavenging percentage was expressed with respect to the control (without the addition of antioxidants) as follows:

% NO scavenging activity = (A_0_ − A_1_)/A_0_ × 100

where A_0_ is the absorbance of reaction mixture + vehicle solvent (control); A_1_ is the absorbance of the reaction mixture (containing the different concentrations of a fraction and Griess reagent).

### 2.5. Column Chromatography of the CaLE Fraction

**Scheme 2:**
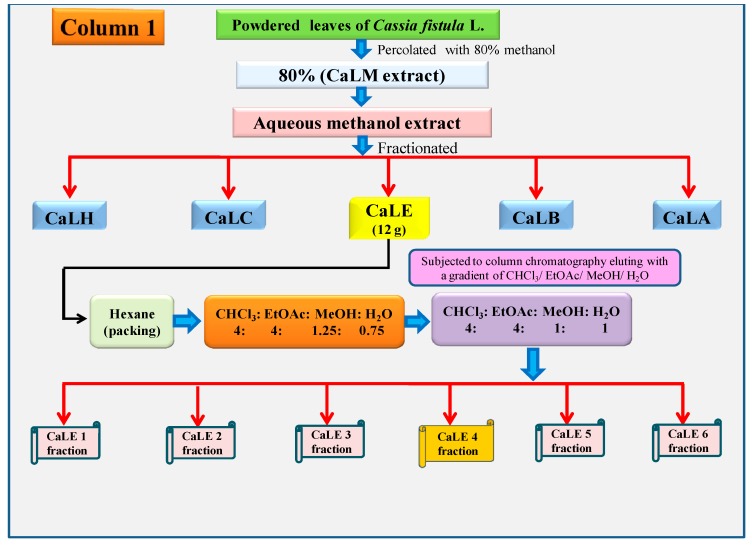
Schematic representation of the isolation of the ‘CaLE4′ subfraction from the CaLE fraction of the *C. fistula* leaves.

**Scheme 3:**
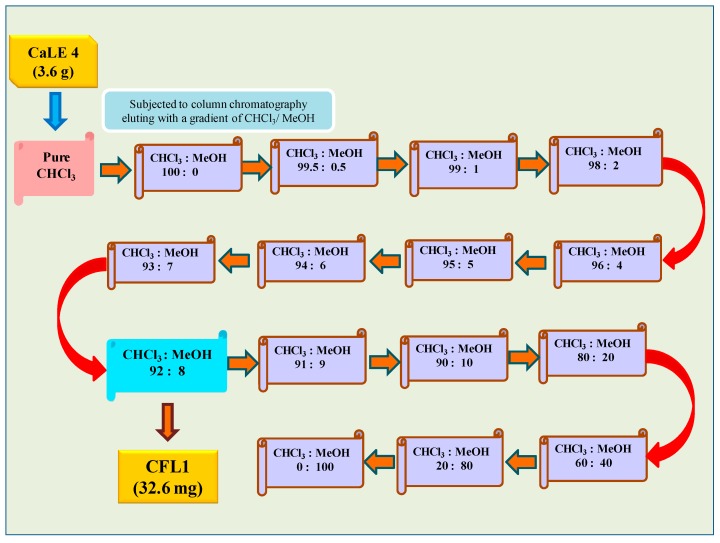
Schematic representation of the isolation of the ‘CFL1′ compound from the ‘CaLE4′ subfraction of the CaLE fraction from the *C. fistula* leaves.

The dried CaLE fraction (12 g) of *C. fistula* leaves was subjected to column chromatography, and the column was packed with chloroform. The slurry was packed in a column containing silica gel and a gradient of CHCl_3_:EtOAc:MeOH:H_2_O (4:4:1.25:0.75) was used as eluent. Then the polarity was increased to CHCl_3_:EtOAc:MeOH:H_2_O (4:4:1:1). About 42 fractions were collected at the gradient of CHCl_3_:EtOAc:MeOH:H_2_O (4:4:1:1), which were pooled, concentrated, and lyophilized based on their TLC. The column chromatography yielded CaLE1 fraction, CaLE2 fraction, CaLE3 fraction, CaLE4 fraction, CaLE5 fraction, and CaLE6 fraction. The subfraction CaLE 4 (3.6 g) (Scheme 2) was further subjected to column chromatography with a gradient elution of CHCl_3_:MeOH (100:0), (99.5:0.5), (99:1), (98:2), (96:4), (95:5), (94:6), (93:7), (92:8), (91:9), (90:10), (80:20), (60:40), (20:80), and (0:100). The fractions collected in CHCl_3_:MeOH was pooled, concentrated, and lyophilized. The precipitates obtained at the gradient of CHCl_3_:MeOH (92:8) yielded 32.6 mg of CFL1 fraction as pure compound (Scheme 3). 

### 2.6. Nuclear Magnetic Resonance (NMR) Spectroscopy

NMR analysis of CFL1 was carried out on a Bruker NMR-500 MHz (Bruker Corporation, Massachusetts, USA) to get ^1^H NMR, ^13^C NMR, and DEPT-135 spectra (Bruker Corporation, Massachusetts, USA). 

### 2.7. FT-IR

FT-IR is used for identifying the organic molecular groups and compounds, functional groups, and cross-links involved, all of which are having characteristics vibrational frequencies in the infra-red range. FTIR of CFL1 was carried out on Agilent-FT-IR technologies.

### 2.8. HPLC Profiling

The quantitative HPLC profiling of fractions from *C. fistula* was carried out using a Shimadzu UHPLC Nexera system (Shimadzu, MA, USA), equipped with a quaternary pump (LC-30AD) and a degasser. The injection volume of the sample was 5 μL. The analytical column used was the Enable C18 column (150 mm × 4.6 mm i.d. × 5 μm p.s.), (Shimadzu, MA, USA) that is also provided with the guard column (10 × 4 mm). For chromatographic analysis, the mobile phase elution was a continuous gradient of solvent A (0.1% acetic acid in water, pH 3) and solvent B (methanol). The setting of gradient program was as mentioned (0–10 min) 30% B, (10–14 min) 65% B, (14–16 min) 80% B, (16–17 min) 40% B, (17–17.50 min) 35% B and (17.50–21 min) 30% B. The total run time was 21 min with a constant flow rate of 1.0 mL/min. The column oven (CTO-10AS) helps to maintain a constant temperature of 25 °C. The photodiode array (PDA) detector (SPD-M20A) (Shimadzu, MA, USA) was used to monitor the chromatogram at 280 nm. The solvent delivery, detection, and data processing were performed with the help of the Labsolutions software (version 5.09, Shimadzu, MA, USA).

### 2.9. Cell Culture 

PC-3 (Human prostate adenocarcinoma), IMR-32 (Human neuroblastoma), MG-63 (Human osteosarcoma), and CHO (Chinese Hamster Ovary) cell lines were procured from the National Centre for Cell Science, NCCS, Pune, India. The cells were checked daily for their proper growth, and the medium was changed periodically. The cells were sub-cultured at the sub-confluent stage. The culturing of PC-3, IMR-32, MG-63, and normal cell line CHO required DMEM (with 10% FBS and antibiotic-antimycotic solution) for their proper growth at 37 °C in a humidified incubator with 5% CO_2_. 

### 2.10. Measurement of Cell Viability

The viability of cells was checked before experiments using the method as described by Militao et al. [16] with minor modifications. For this, the cells were washed with PBS (pH 7.4) followed by trypsinization and finally centrifuged for 2–5 min at 1500 rpm. The cell suspension was made by suspending the cell pellet in fresh media and stained with 0.4% trypan blue dye (prepared in PBS) to determine the number of viable cells (not stained) and non-viable cells (stained). After determining viability, cells were used for performing different experiments.

### 2.11. MTT Assay

The cytotoxic potential of the fractions from *Cassia fistula* L. was determined by using MTT assay as described by the method of Mickisch et al. [17] with minor modifications. In this experiment, cells were seeded in 96 well microplates at the concentration of 8 × 10^3^ cells/0.1 mL and incubated to allow cell attachment. After 24 h, cells were treated with the different concentrations of the extracts/fractions using the serial dilutions method. On the completion of a total 48 h, 20 µL of 3-(4,5-dimethylthiazol-2-yl)-2,5-diphenyltetrazolium bromide (MTT) was added to each well and the ability of viable cells to reduce it into insoluble purple-colored formazan was measured and the cells were further incubated for 3 h. After this, the supernatant containing the MTT solution was removed from each well, and the intracellular MTT formazan was dissolved in 100 µL of dimethyl sulfoxide (DMSO). Finally, the decrease in absorbance was measured at 570 nm using a multiwell plate reader (BioTek Synergy HT, Winooski, USA).

Cell viability = (OD_570_ of treated sample/OD_570_ of untreated control) × 100

The growth inhibition percentage was expressed by using the following equation

% Growth inhibition = 100% − %age cell viability

### 2.12. Morphological Studies using Confocal Microscopy

MG-63 cells were seeded in a six-well plate at a density of 3 × 10^5^ cells/well in which a coverslip was placed in each well. After 24 h, cells adhered and were then treated with various concentrations of the CFL1. After 24 h, the morphology of the cells was observed under an inverted microscope. For visualizing nuclear morphology viz. DNA fragmentation, nuclear condensation, and apoptotic cell death, cells were stained using Hoechst staining [18]. The media was discarded, and cells were washed twice with PBS and then fixed with 0.4% paraformaldehyde at ambient temperature for 20 min. Again, the cells were washed thrice with PBS and stained by adding 5 µL of Hoechst 33342 (1 mg/mL) staining solution in the dark at 37 °C for 30 min. On the slides, anti-fading reagent (Fluoromount, Sigma, St. Louis, MI, USA) was poured over the center and then a glass coverslip was gently put on it. Finally, the slides were observed under a Nikon A1R Laser Scanning Confocal Microscope system (Nikon Corporation, Tokyo, Japan) using the NIS Elements AR analysis software version 4.11.00 (Nikon Corporation, Tokyo, Japan) for assessing the nuclear morphology of the cells. 

### 2.13. Morphological Studies using scanning Electron Microscopy (SEM)

The scanning electron microscopy of the fraction from *C. fistula* was assessed, as mentioned by the protocol of Rello et al. [19]. MG-63 cells at a density of 2 × 10^5^ cells/well were seeded in a six-well plate containing polylysine-coated coverslip in each well for adherence for 24 h. Thereafter, the cells were treated with various concentrations of CFL1. After 24 h, the media was discarded and the cells were washed twice with PBS. This was followed by the fixation of cells with 0.25% glutaraldehyde followed by dehydration with a series of graded ethanol (30%, 50%, 70%, and 90% ethanol). Finally, a drop of hexamethyldisilazane was added on a coverslip for 2–3 min and then removed for complete dehydration, and the coverslip was mounted on conventional stubs by placing them carefully in the upright position on the double-adhesive carbon tape. The sample was coated with gold using a sputter coater and observed under the scanning electron microscope (Carl Zeiss, Model No. EVOLS10, Jena, Germany).

### 2.14. Flow Cytometric Studies

#### 2.14.1. Reactive Oxygen Species Generation Analysis

The reactive oxygen species formation was analyzed as described by the method of Deeb et al., [20]. The MG-63 cells were cultured at a density of 5 × 10^5^ cells/well in a six-well plate for 24 h. After adherence, cells were treated with various concentrations of CFL1 for another 24 h. After treatment, the media was decanted off, and the cells were washed with PBS. Further, before the termination of experiments, cells were incubated with 2′,7′-dichlorofluorescein diacetate (DCFH-DA) (5 µg/mL) for 30 min. Then the cells were harvested in 15 mL falcon tubes and centrifuged for 5 min at 1500 rpm. The cell pellet obtained was washed twice with PBS and centrifuged again. Finally, the cell pellet was suspended in 500 μL of PBS. Immediately, the cell suspension was analyzed by flow cytometry (BD Accuri C6 Flow Cytometer, BD Biosciences, San Jose, CA, USA) using the BD Accuri software (version 1.0.264.21). The analysis of cells was done using the FL-1 channel (Emission λ 535 nm; excitation λ 488 nm). 

#### 2.14.2. Mitochondrial Membrane Potential (Δ*Ψm*) Analysis

The mitochondrial membrane potential (Δ*Ψm*) of the fraction was analyzed as described by the method of Pajaniradje et al. [21]. The MG-63 cells at a density of 5 × 10^5^ cells/well were seeded in six-well plates for 24 h. After adherence, the cells were treated with various concentrations of the CFL1 for 24 h. After treatment, the media was discarded, and the cells were washed with PBS. Then, these cells were incubated with Rhodamine-123 (10 µg/mL) for about 30 min in the dark. Finally, the cells were harvested in 15 mL Falcon tubes and centrifuged for 5 min at 1500 rpm. After centrifugation, the supernatant was decanted, and the cell pellet obtained was washed twice with PBS. The cells were centrifuged again, and finally, the cell pellet obtained was suspended in 500 μL of PBS. Immediately, the cell suspension was analyzed by flow cytometry (BD Accuri C6 Flow Cytometer, BD Biosciences) using the BD Accuri software (version 1.0.264.21, San Jose, CA, USA). The analysis of cells was done using the FL-1 channel (Emission λ 535 nm; excitation λ 488 nm). 

#### 2.14.3. Cell-Cycle Phase Distribution Analysis

The cell cycle phase distribution analysis in MG-63 cells treated with CFL1 was performed using the BD Cycletest plus DNA Kit (BD Biosciences). The cell culture at the density of 5 × 10^5^ cells were cultured in each well of 6 well plates was allowed for attachment. On completion of 24 h, MG-63 cells were treated with different concentrations of the CFL1. After treatment for 24 h, both the floating as well as adhered cells were collected in the falcon tubes (15 mL) followed by centrifugation for 5 min. The pellet of cells obtained was washed two times with PBS and centrifuged at 1500 rpm. Then 70% ethanol solution (1 mL) was added to the cell pellet for fixation, and the cell pellet was kept for 2–3 h at –20 °C. After fixation, the suspension of cells formed was washed two times with PBS. A total of 250 µL of solution A (trypsin buffer) was added in each tube and incubated for 10 min at room temperature followed by the addition of 200 µL of solution B (trypsin inhibitor and RNase buffer). After incubation, 200 µL of cold solution C (Propidium iodide stain solution) was added and incubated for 60 min on ice in the dark. The stained cells were estimated with the help of the BD Accuri software by flow cytometry (BD Accuri C6 Flow Cytometer, BD Biosciences, San Jose, USA). 

#### 2.14.4. Assessment of Apoptosis by Annexin V-FITC/PI Double Staining

Apoptosis was also investigated in MG-63 cells treated with CFL1 using fluorescein isothiocyanate (FITC)-conjugated Annexin V/PI assay (BD Pharmingen Annexin V FITC apoptosis detection kit, Biosciences). The cells at the density of 5 × 10^5^ were cultured in each well of the six-well plates and allowed time for adherence. On completion of 24 h, cells were treated with different concentrations of the sample. After treatment, adhered as well as floating cells were collected in 15 mL tubes and centrifuged for 5 min. Then the pellets of cells obtained were washed two times with PBS and centrifuged. After centrifugation, the cell pellet was suspended in 100 µl of binding buffer for 15 min followed by the addition of Annexin V-FITC conjugate (5 µL) and Propidium iodide (5 µL) and incubated at room temperature for 20 min in the dark. Finally, to this cell suspension, the binding buffer was added. The stained cells were analyzed by flow cytometry (BD Accuri C6 Flow Cytometer, BD Biosciences) using the BD Accuri software. 

### 2.15. Western Blotting

The protocol for Western Blotting was used from the Bio-Rad protocols.

#### 2.15.1. Sample Preparation

For the isolation of proteins, MG-63 cells (2 × 10^6^) were cultured in the Petri plates and were kept for complete adherence to the matrix for 24 h at 37 °C. Cells were treated with different concentrations of the CFL1 and incubated for 24 h. After incubation, cells were scraped out with the help of a cell scraper and centrifuged at 2500 rpm for 5–6 min, then the media was decanted, and the pellet was washed twice with ice-cold PBS. Further, the cell pellet was mixed with 150 µL of RIPA (Radioimmunoprecipitation assay) buffer and incubated for 25 min on ice, centrifuged at 12,000 rpm for 25 min. Then the cell pellet was discarded, and supernatant (containing proteins) was collected.

#### 2.15.2. Protein Quantification by Bradford Method

For the estimation of proteins, 10 standard concentrations (1–10 µg) of BSA (1 mg/mL stock solution) and then 5 µL of protein sample was added in the 96-well plate. Then, the final volume was raised by the addition of 20 µL of distilled water to each well. Then Bradford reagent (200 µL) was added to each sample and incubated for 10–15 min at room temperature in the dark. The absorbance was measured at 595 nm, and protein concentrations were calculated from the standard BSA curve. 

#### 2.15.3. Sample Loading/Gel Electrophoresis/Transfer to PVDP

The resolving gel solution (12%) was prepared and poured in the casting plates. After the polymerization of the resolving gel, stacking gel was prepared and poured in the casting plates, a comb was then inserted to form the loading wells. Protein lysate (40 µg) were prepared with loading dye by heating and then boiled at 100 °C for 4–5 min and finally loaded to the gel. The gel was run at 70 V and after resolving the gel, the blot was transferred onto a PVDF (Polyvinylidene difluoride) membrane (MDI) using the wet transfer (Bio-Rad assembly, California, USA). 

#### 2.15.4. Antibody Incubation/Imaging

Further, membrane blocking was performed with skimmed milk (5% in TBST (Tris-buffered saline, 0.1% Tween 20)) for 2 h at room temperature and probed with rabbit monoclonal Bcl-xl (1:1000), p-Akt (1:2000), and p-GSK-3β (1:1000) antibodies (Cell Signaling Technology) at 4 °C for overnight. The membrane was washed three times for 20 min each with TBST and incubated with the secondary antibody anti-rabbit HRP labeled for 2–3 h. The immunoreactive bands obtained were analyzed with the help of ECL Plus Western blot detection system (Bio-Rad, California, USA) using LAS 4000 (GE Biosciences). The expression of β-actin (endogenous control) was used on the same membrane. The final expression of each protein was calculated by using image-J software (version 1.52e, NIH, USA).

### 2.16. RT-PCR

Real-Time PCR experimental data were analyzed according to the method given by Schmittgen and Livak [22]. For the isolation of RNA, MG-63 cells (2 × 10^6^) were seeded in the Petri plates and were kept for complete adherence at 37 °C for 24 h. Cells were treated with different concentrations of the CFL1 and incubated for another 24 h. After incubation, the cells were washed with ice-cold PBS and lysed directly in a culture dish by adding Trizol Reagent (1 mL), and the cells were scraped with scraper or pipette tip followed by incubation for 5 min. Then add 200 µL chloroform and vortex vigorously for 15 s, centrifuged at 12,000 rpm at 4 °C for 20 min. The mixture was separated into the lower organic and upper aqueous phase that contains RNA. The upper aqueous phase was transferred into a fresh tube and added 0.5 mL of 70% ethanol to precipitate the RNA and incubated for 10 min followed by centrifugation at 12,000 rpm at 4 °C for 20 min. The supernatant was removed and the RNA pellet was collected. Then RNA pellet was dried for 5–10 min on a dry bath followed by the addition of DEPC-treated water to dissolve RNA. Finally, the OD of RNA was measured at 260 nm and 280 nm. Further, equal amounts of RNA of different concentrations were used for making cDNA using the cDNA synthesis kit (Bio-rad). The RT-PCR reaction was carried out on Applied Biosystems to check the relative expression using SYBR (Bio-rad). Relative quantification of each gene expression was performed using the comparative threshold cycle method (ΔΔCT). For each gene of interest, β-actin served as control. Each Ct value was normalized by the Ct value of β-actin RNA. For each gene, the relative gene expression was defined as 2-ΔΔCt, and final gene expression was expressed as 2-ΔΔCt ± SEM. All reactions were performed in triplicate and the primer sequences are shown in Table 1.

One reaction mixture for RT-PCR reaction contains reagents as SYBR (5 µL), cDNA (1 µg), nuclease-free water (variable), primer (Forward + Reverse, 0.4 µL). RT-PCR cycling conditions for CDK2, p53, Caspase 8, and β-catenin are 95 °C (10 min.), 95 °C (15 sec), annealing temperature (1 min.), 72 °C (1 min); step 2–4 (40 cycles), and melt curve (according to instrument protocol).

### 2.17. Statistical Analysis

The statistical significance of the data was estimated by one-way analysis of variance ANOVA (F-test). The difference among the means was further analyzed by high range statistical domain (HSD) using Tukey’s test. All the values were presented as Mean ± Standard Error in triplicate for all the experiments and the regression equation was carried out with the help of Microsoft Excel. The values statistically significant at 5% level of significance were assessed at probability *p* ≤ 0.05.

## 3. Results 

### 3.1. Antioxidant Activity

#### 3.1.1. Ferric ion Reducing Antioxidant Power (FRAP) Assay

Among all the extract/fractions of *Cassia fistula* leaves, the CaLE fraction and an isolated CFL1 fraction showed potent electron-donating capacity and reduction potential with the maximum absorbance value of 0.855 and 0.887 in Ferric ion reducing antioxidant power (FRAP) assay. The reducing ability of the fraction was revealed by the significant increase in absorbance at 700 nm, with an increase in the concentration (50–800 µg/mL) (Table 2).

#### 3.1.2. ABTS^•+^ Scavenging Assay 

**Figure 1:**
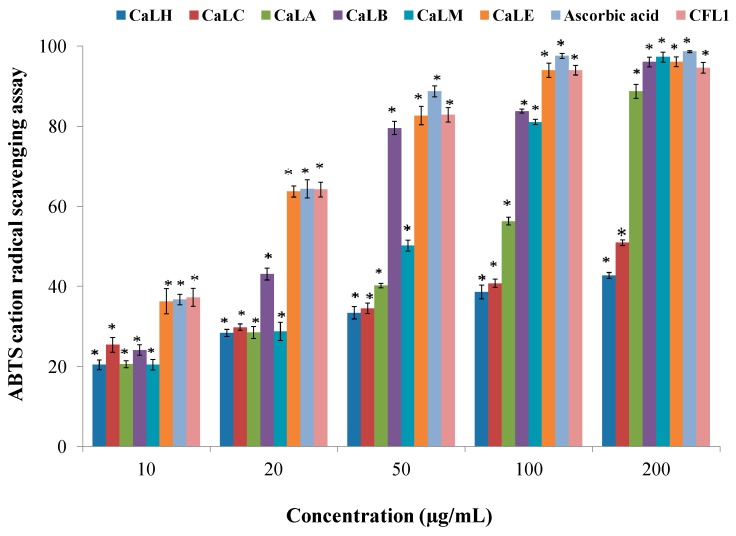
ABTS (2,2’-azino-bis(3-ethylbenzothiazoline-6-sulfonic acid)) cation radical scavenging effect of extract/fractions from *C. fistula* and standard ascorbic acid (values are represented as percent of control). * indicates statistically significant difference at (*p* ≤ 0.05) (For each fraction *n* = 18).

The CaLE and isolated CFL1 fractions exhibited strong scavenging of ABTS radical cations with an IC_50_ of 13.2 µg/mL and 12.66 µM in comparison with other fractions (Figure 1).

#### 3.1.3. Nitric Oxide Radical Scavenging Assay 

**Figure 2:**
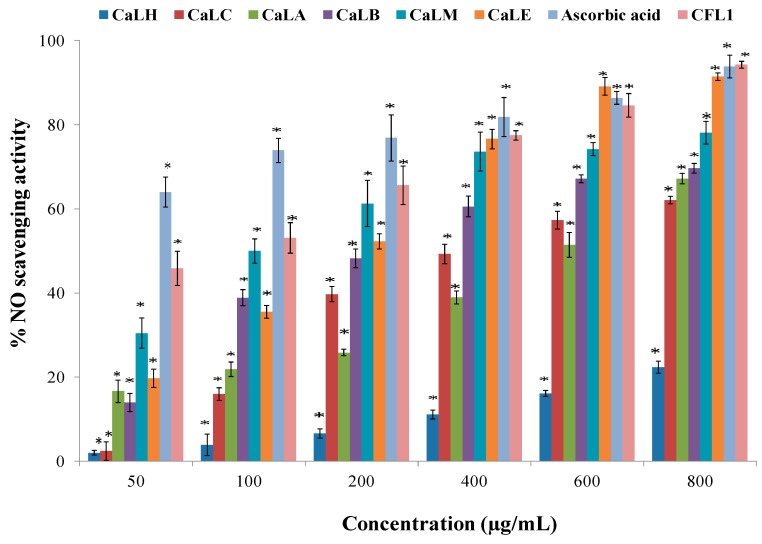
Nitric oxide radical scavenging effects from extract/fractions from *C. fistula* and standard ascorbic acid (values are represented as percent of control). * indicates a statistically significant difference at (*p* ≤ 0.05) (For each fraction *n* = 18).

The CaLE fraction and an isolated CFL1 fraction demonstrated a significant scavenging potential against nitric oxide radicals with an inhibition percentage of 91.44% and 94.26%. However, other fractions showed moderate scavenging activity in the following order: CaLM (78.07%) > CaLB (69.63%) > CaLA (67.18%) > CaLC (62.06%), least effective being CaLH (22.34%) (Figure 2). 

### 3.2. Characterization and Structure Elucidation of CFL1 Fraction 

**Figure 3:**
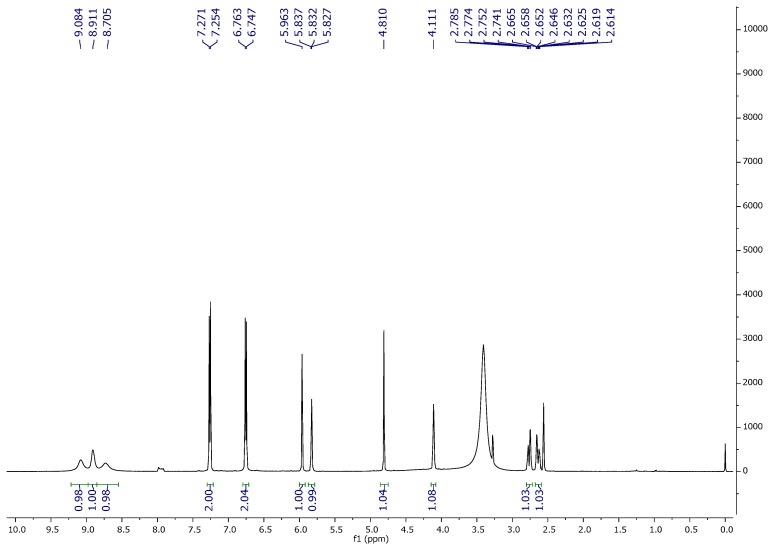
^1^H NMR of the CFL-1 (Epiafzelechin) fraction of *C. fistula* leaves.

**Figure 4:**
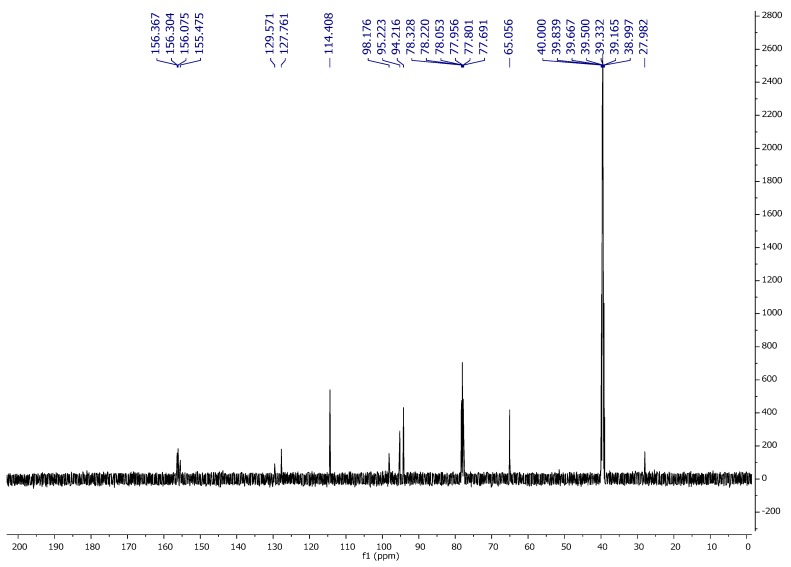
^13^C NMR of the CFL-1 fraction of *C. fistula* leaves.

**Figure 5:**
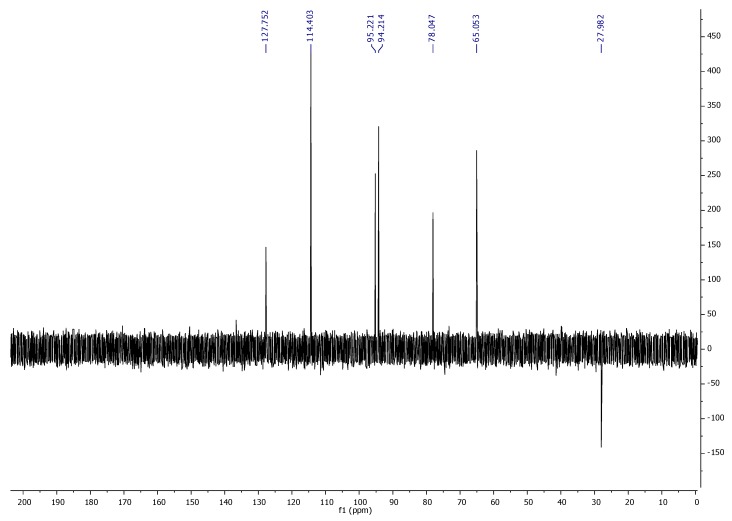
DEPT (Distortionless enhance by polarization transfer)-135 of the CFL-1 fraction of *C. fistula* leaves.

**Figure 6:**
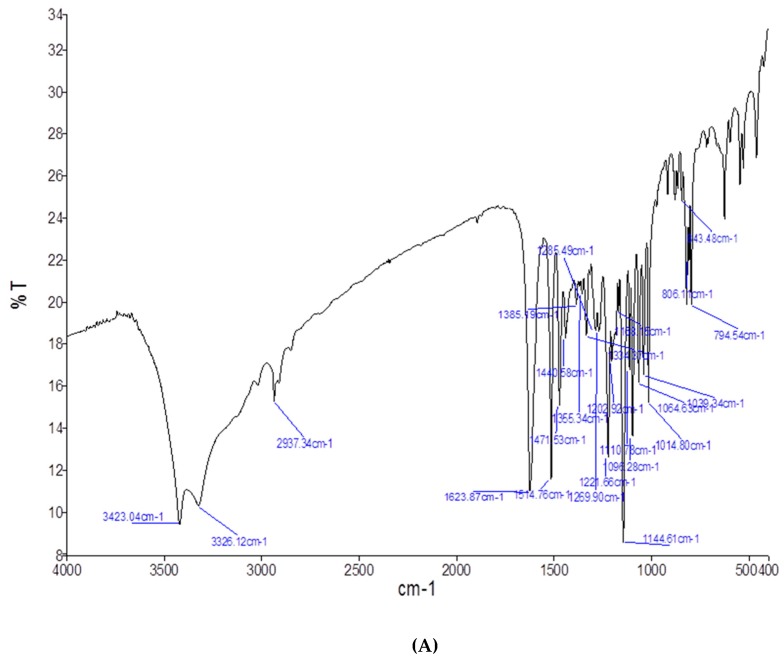

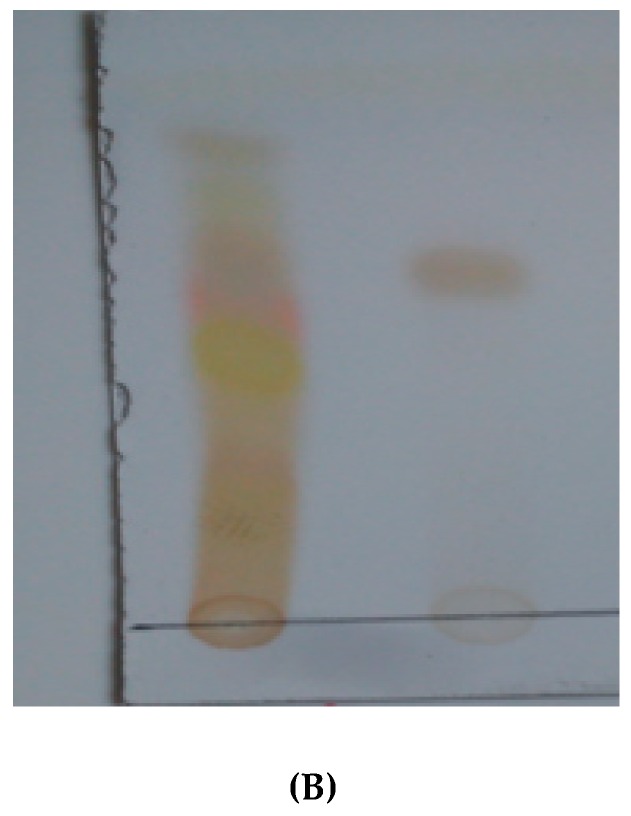
(**A**) FT-IR (Fourier-transform infrared) spectrum of the CFL-1 fraction of *C. fistula* leaves; (**B**) Plate I: thin layer chromatography of fractions from *C. fistula* (Solvent system-CHCl_3_:MeOH 9:1).

CFL1 was obtained as a light brown colored powder. ^1^H NMR (DMSO-D_6_ + CDCl_3_, 500 MHz) *δ* 9.08 (1H, br s, Ar-OH), 8.91 (1H, br s, Ar-OH), 8.70 (1H, br s, Ar-OH), 7.26 (2H, d, *J* = 8.5 Hz, Ar-H), 6.76 (2H, d, *J* = 8.0 Hz, Ar-H), 5.96 (1H, s, Ar-H), 5.82–5.83 (1H, m, Ar-H), 4.81 (1H, s, aliphatic-CH), 4.11 (1H, s, aliphatic-CH), 2.74–2.78 (1H, dd, *J* = 16.5, 3.0 Hz, aliphatic-CH), 2.61-2.66 (1H, m, aliphatic-CH) (Figure 3). ^13^C NMR (DMSO-D_6_ + CDCl_3_, 125 MHz) *δ* 156.36, 156.30, 156.0, 155.4, 129.5, 127.7, 114.4, 98.1, 95.2, 94.2, 78.0, 65.0, 27.9 (Figure 4). DEPT 135 NMR (125 MHz, CDCl_3_) *δ* 127.7 (CH), 114.4 (CH), 95.2 (CH), 94.2 (CH), 78.0 (CH), 65.0 (CH), 27.9 (CH_2_) (Figure 5). FT-IR spectrum (ATR): ν_max_ [cm^−1^] = 3423, 3326, 2937, 1623, 1514, 1471, 1440, 1385, 1355, 1285, 1221, 1202, 1110, 1096, 1064, 1014 (Figure 6A). The thin-layer chromatography (TLC) of the compound gave a single spot of light purple color when exposed to iodine (Plate I) (Figure 6B). Therefore, on the basis of spectroscopic studies, the molecular formula of the compound was C_15_H_14_O_5_^+^. Thus, on the basis of the above spectral data and in comparison with previously known spectral values, the structure of fraction CFL1 was assigned as Epiafzelechin [23]. 

### 3.3. HPLC Profiling

**Figure 7:**
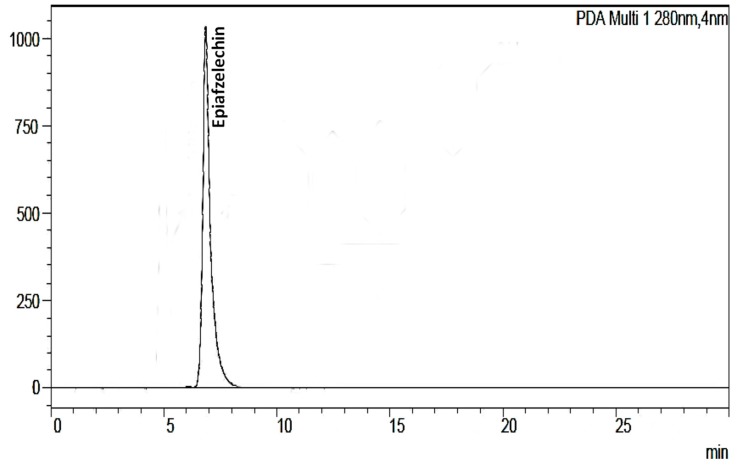
The HPLC (High-Performance Liquid Chromatography) chromatogram of Epiafzelechin (CFL1 fraction) isolated from *C. fistula* leaves.

HPLC analysis of Epiafzelechin (CFL1 fraction) revealed the presence of Epiafzelechin as a single peak (Figure 7). 

### 3.4. Antiproliferative Activity

**Figure 8:**
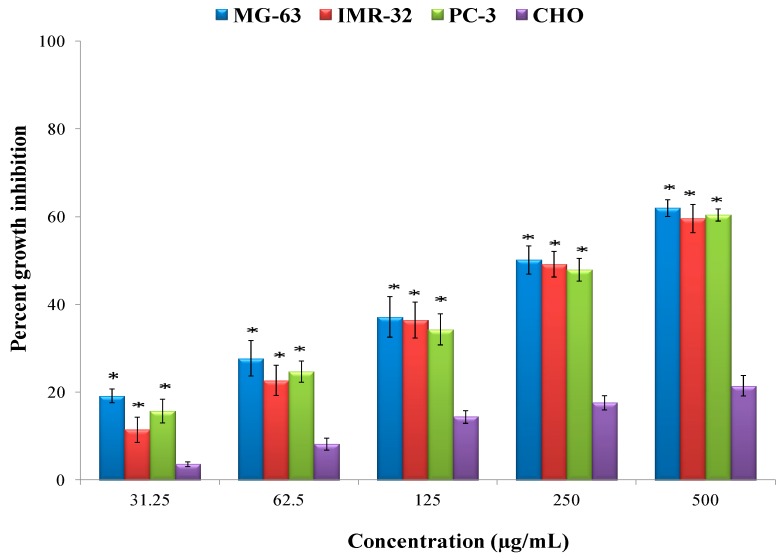
Antiproliferative activity of the CaLE fraction from *C. fistula* on different cell lines in the MTT (3-(4,5-dimethylthiazol-2-yl)-2,5-diphenyltetrazolium bromide) assay (*n* = 60). * indicates a statistically significant difference at (*p* ≤ 0.05).

**Figure 9:**
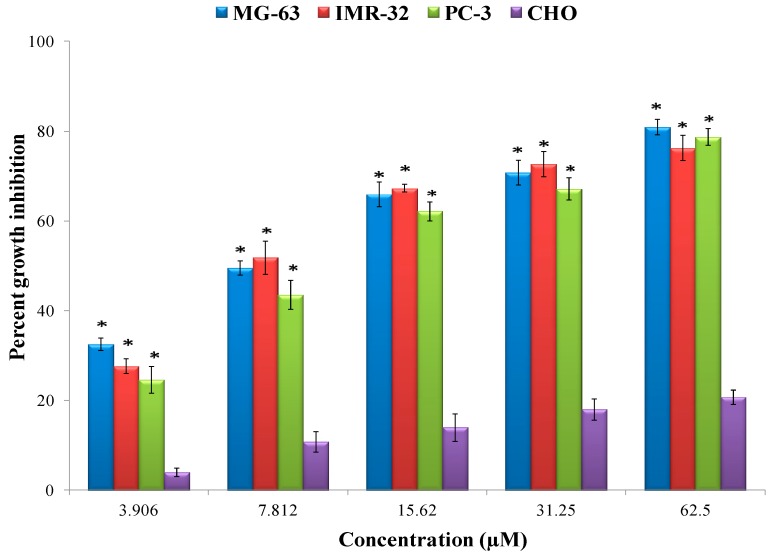
Antiproliferative activity of Epiafzelechin isolated from *C. fistula* on different cell lines in the MTT assay (*n* = 60). * indicates statistically significant difference at (*p* ≤ 0.05).

The CaLE fraction induced the effective cytotoxic effects against MG-63, IMR-32, and PC-3 cancer cell lines with a GI_50_ of 249.9, 278.4, and 286.2 µg/mL, respectively (Figure 8). The Epiafzelechin (CFL1) isolated from the CaLE fraction of *Cassia fistula* leaves induced a stronger cytotoxic potential towards the MG-63 cancer cell line with the GI_50_ value of 8.73 μM. The fraction was found to exhibit an antiproliferative effect against IMR-32 and PC-3 with the GI_50_ values of 9.15 and 11.89 μM, respectively. The fractions were non-cytotoxic against the normal cell line, CHO. CFL1 showed maximum antiproliferative activity against MG-63, which was further probed for its mechanism of action (Figure 9). 

### 3.5. Morphological Studies using Confocal Microscopy 

**Figure 10:**
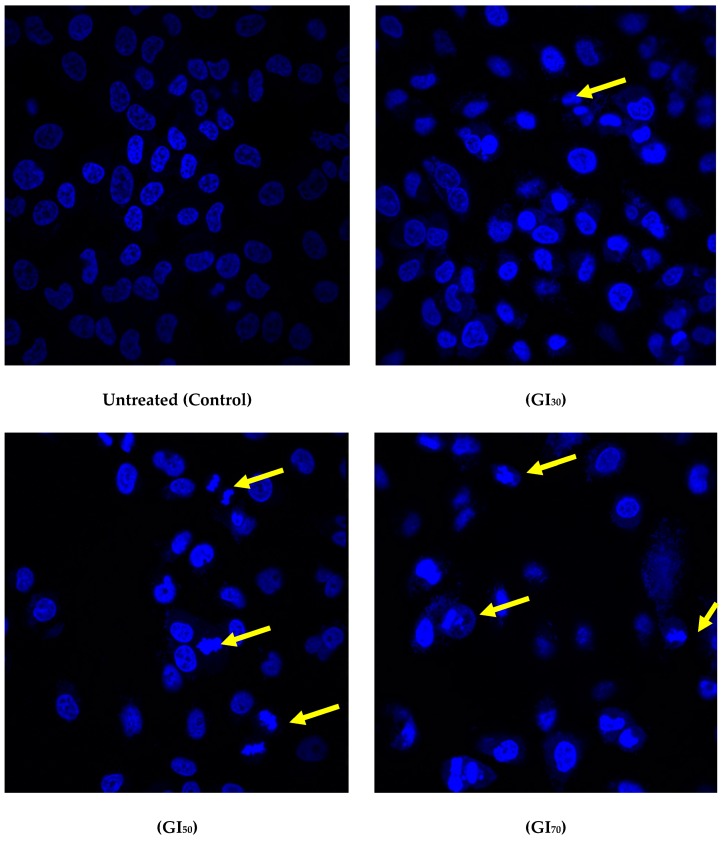
Confocal micrographs of Hoechst 33342 stained MG-63 cells treated with Epiafzelechin from *C. fistula* for 24 h. Arrows show nuclear condensation and formation of apoptotic bodies at a magnification 60× oil immersion objective lens.

The treatment of MG-63 cells with different concentrations of Epiafzelechin (GI_30_ = 2.69 µM, GI_50_ = 8.73 µM and GI_70_ = 28.41 µM) exhibited morphological changes like nuclei showed fragmentation and chromatin condensation. However, in the case of untreated control cells, an intact nucleus was observed without any sign of fragmentation and chromatin condensation (Figure 10).

### 3.6. Morphological Studies using Scanning Electron Microscopy (SEM) Studies 

**Figure 11:**
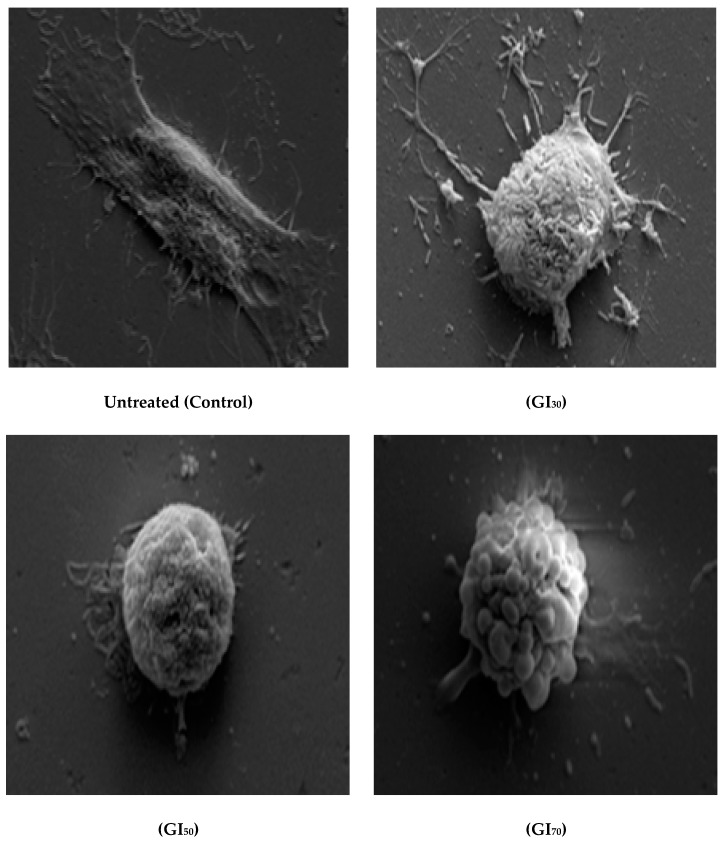
Scanning Electron Micrographs (SEM) of MG-63 cells before and after treatment with different concentrations of Epiafzelechin from *C. fistula* (Magnification of 14 KX, 14,000 times).

The SEM studies revealed that the MG-63 cells treated with different concentrations (GI_30_, GI_50,_ and GI_70_) of Epiafzelechin showed various apoptotic features like cell shrinkage, membrane blebbing, and formation of apoptotic bodies. In the case of untreated control cells, intact cell morphology was observed (Figure 11).

### 3.7. Flow Cytometric Analysis

#### 3.7.1. Measurement of A Generation of Intracellular Reactive Oxygen Species (ROS) 

**Figure 12:**
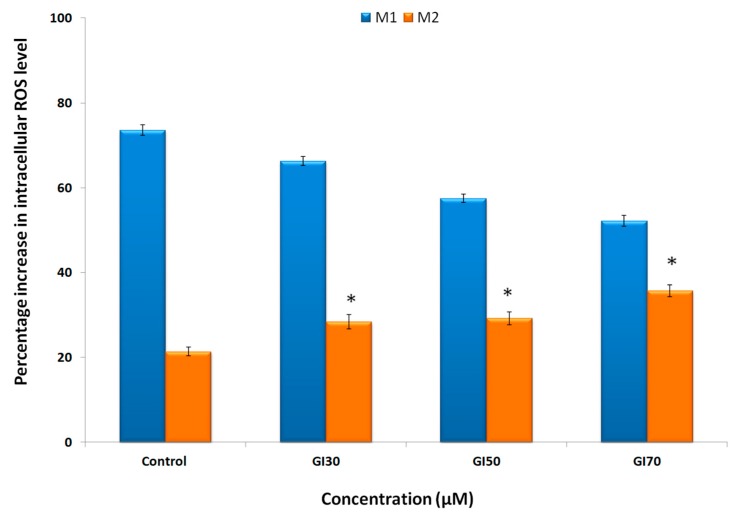
Effect of Epiafzelechin from *C. fistula* on the generation of intracellular reactive oxygen species (ROS) in MG-63 cells (24 h). 2′,7′-dichlorofluorescein diacetate (DCFH-DA) was used to stained the cells, 20,000 events were acquired and the gated population analyzed using flow cytometer. M1 represents intact cell population and M2 represents cells with accumulation of intracellular ROS. * indicates a statistically significant difference at (*p* ≤ 0.05).

The generation of Reactive Oxygen Species (ROS) plays a crucial role in the induction of apoptosis and depolarizing the mitochondria. The formation of intracellular ROS was measured by 2′,7′-dichlorofluorescein diacetate (DCFH-DA)—a non-fluorescent cell membrane permeable probe that is cleaved by ROS/non-specific cellular esterases and oxidized in the presence of peroxidases and H_2_O_2_ into the fluorescent 2′,7′-dichlorofluorescein (DCF). The intracellular ROS generation in control and Epiafzelechin treated MG-63 cancer cells was assessed by FL-1 channel (Excitation 488 nm; Emission 535 nm) of the BD Accuri C6 flow cytometry followed by staining with DCFH-DA and their representative histograms, as given in Figure 12.

The treatment of cancer cells with different concentrations of Epiafzelechin, i.e., GI_30_, GI_50_, and GI_70_ concentrations showed enhancement in the ROS generation by 28.4%, 29.2%, and 35.7%, respectively as compared to control cancer cells (21.4%), as shown in Figure 12. The cells treated with Epiafzelechin showed an increase in the DCF fluorescence dose-dependently against cancer cells, which indicates the generation of ROS and, in turn, indicates the apoptosis-inducing ability.

#### 3.7.2. Measurement of Mitochondrial Membrane Potential (Δ*Ψm*) 

Mitochondria play an important role in the intrinsic pathway of apoptosis due to the involvement of the membrane permeability and membrane potential (*Ψm*) in the release of certain apoptogenic factors like apoptosis-inducing factor (AIF) and cytochrome c into the cell cytoplasm. Rhodamine 123—a fluorescent probe was used to determine the changes in the mitochondrial membrane potential on the treatment of MG-63 cancer cells with different concentrations (GI_30_, GI_50,_ and GI_70_) of Epiafzelechin. 

**Figure 13:**
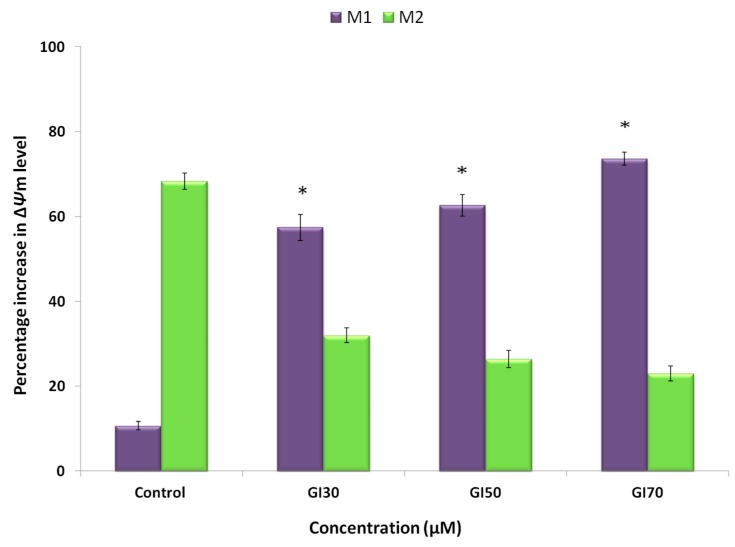
Effect of Epiafzelechin from *C. fistula* on the disruption of mitochondrial membrane potential (Δ*Ψ*m) in MG-63 cells detected by staining with Rhodamine-123. Twenty-thousand events were acquired and the gated population analyzed using flow cytometry. M1 represents cells with the disruption of Δ*Ψ*m and M2 represents the intact cells. * indicates a statistically significant difference at (*p* ≤ 0.05).

It was observed that MG-63 cancer cells became more susceptible to mitochondrial membrane depolarization and showed an increase of 57.4%, 62.6%, and 73.6% on the exposure to Epiafzelechin different concentration (GI_30_, GI_50,_ and GI_70_) respectively in comparison to control of 10.7%. The flow cytometric analysis demonstrated that the treatment with Epiafzelechin showed a dose-dependent attenuation in the membrane potential of MG-63 cells after 24 h, as depicted in Figure 13. 

#### 3.7.3. Cell Cycle Distribution

**Figure 14:**
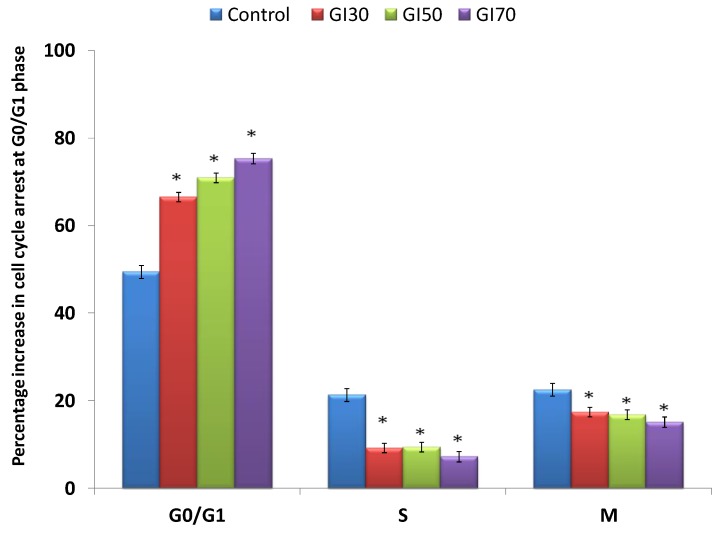
The treatment of Epiafzelechin from *C. fistula* (24 h) induced cell cycle arrest at the G_0_/G_1_ phase in MG-63 cells detected by the cell cycle analysis kit. Ten-thousand events were acquired and the gated cell population analyzed using flow cytometry. * indicates a statistically significant difference at (*p* ≤ 0.05).

In the cell cycle distribution analysis, Epiafzelechin was found to increase the accumulation of the cell population at the G_0_/G_1_ phase of the cell cycle in a dose-dependent manner MG-63 cancer cells. The treatment of Epiafzelechin caused 75% arresting of cells in the G_0_/G_1_ phase at the maximum tested concentration (GI_70_) as compared to the untreated control, as depicted in Figure 14.

#### 3.7.4. Annexin V-FITC/PI Double Staining

**Figure 15:**
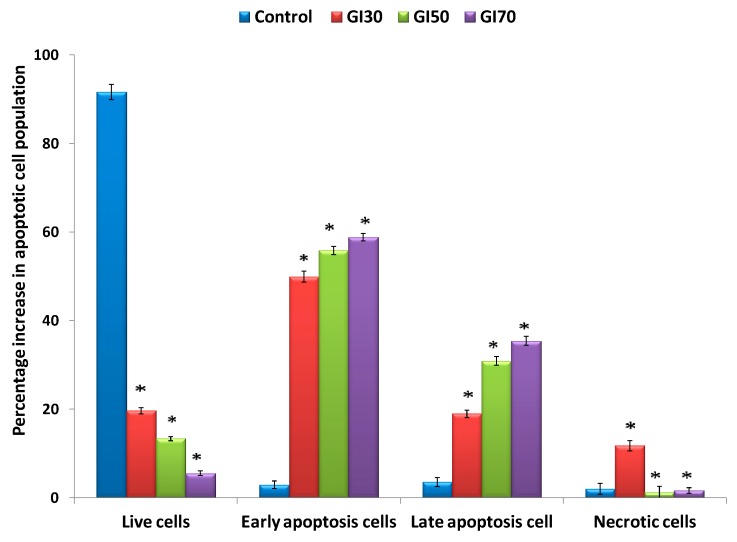
Effect of Epiafzelechin from *C. fistula* on cell apoptosis as detected by Annexin V-FITC/PI (fluorescein isothiocyanate/Propidium iodide) double staining in MG-63 cells indicates the percentage of live cells (Lower left quadrant), early apoptotic cells (Lower right quadrant), late apoptotic cells (Upper right quadrant), necrotic cells (Upper left quadrant). * indicates a statistically significant difference at (*p* ≤ 0.05).

The percentage of early and late apoptotic cell population rates increased in Epiafzelechin treated MG-63 cells when compared with untreated control cells (Figure 15). The results demonstrated that Epiafzelechin could significantly inhibit the growth of human osteosarcoma MG-63 cells through the induction of apoptosis in a dose-dependent manner. 

### 3.8. Western Blotting 

**Figure 16:**
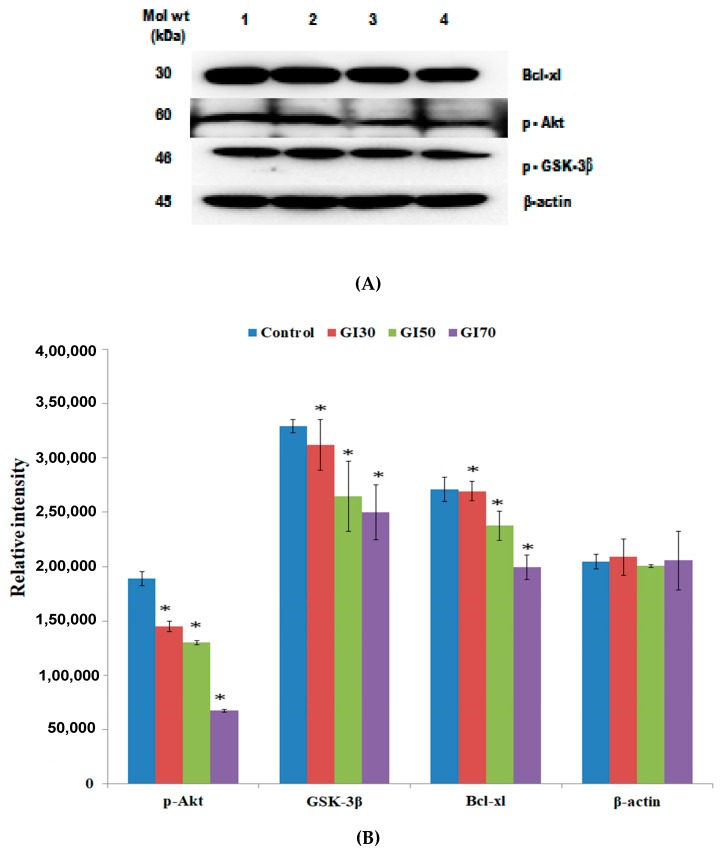
(**A**) Effect of Epiafzelechin from *C. fistula* on the expression level of p-Akt (Protein kinase B), p-GSK-3β (Glycogen synthase kinase-3 beta), and Bcl-xl (B-cell lymphoma-extra large) proteins in MG-63 cells as detected using Western blotting. Band density was measured and normalized to that of β-actin. (Lane 1: control; Lane 2: GI-30 of Epiafzelechin; Lane 3: GI-50 of Epiafzelechin; Lane 4: GI-70 of Epiafzelechin). (**B**) Histograms showing densitometric analysis of Bcl-xl, p-Akt and p-GSK-3β proteins bands in western blotting in Epiafzelechin treated and control cells. Values are expressed as mean ± SE. * indicates statistically a significant difference at (*p* ≤ 0.05).

Epiafzelechin (GI_30_, GI_50_, and GI_70_ concentration) treated MG-63 cells were studied for the expression of p-Akt, p-GSK-3β, and Bcl-xl proteins in Western blotting. The results obtained demonstrated a downregulation in the phosphorylation of Akt and GSK-3β, as indicated by western blot. The protein expression level of anti-apoptotic protein Bcl-xl was downregulated when treated with Epiafzelechin in comparison to controls (Figure 16). 

### 3.9. RT-PCR Analysis

**Figure 17:**
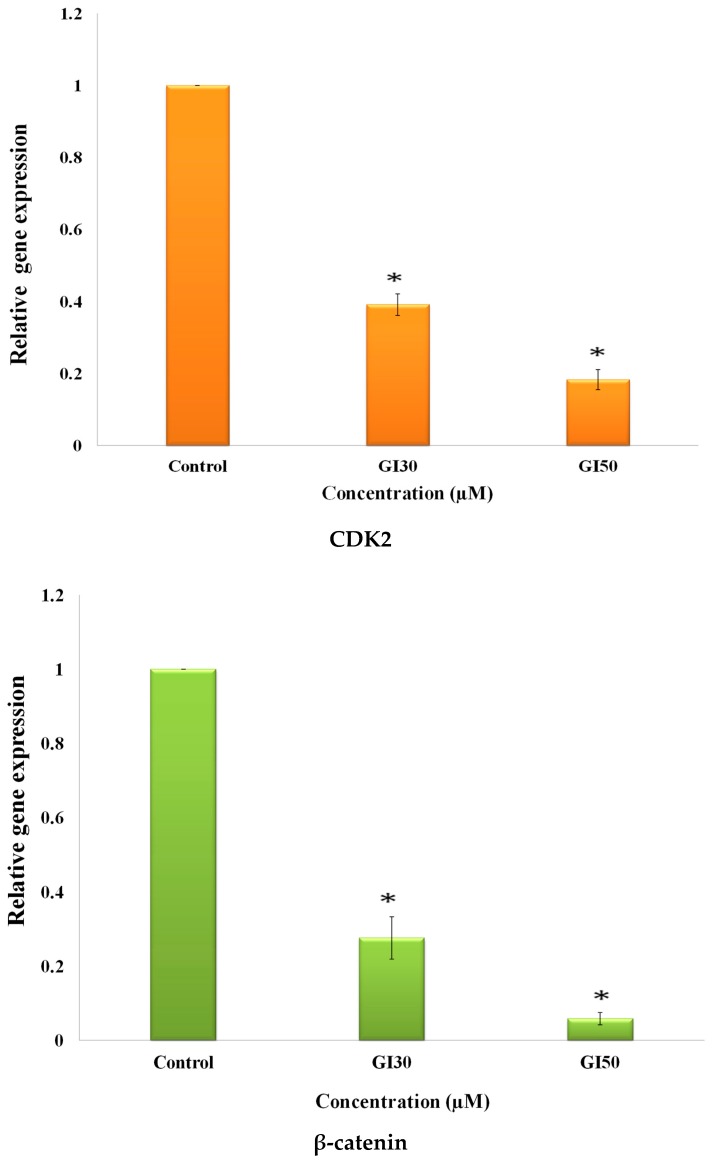

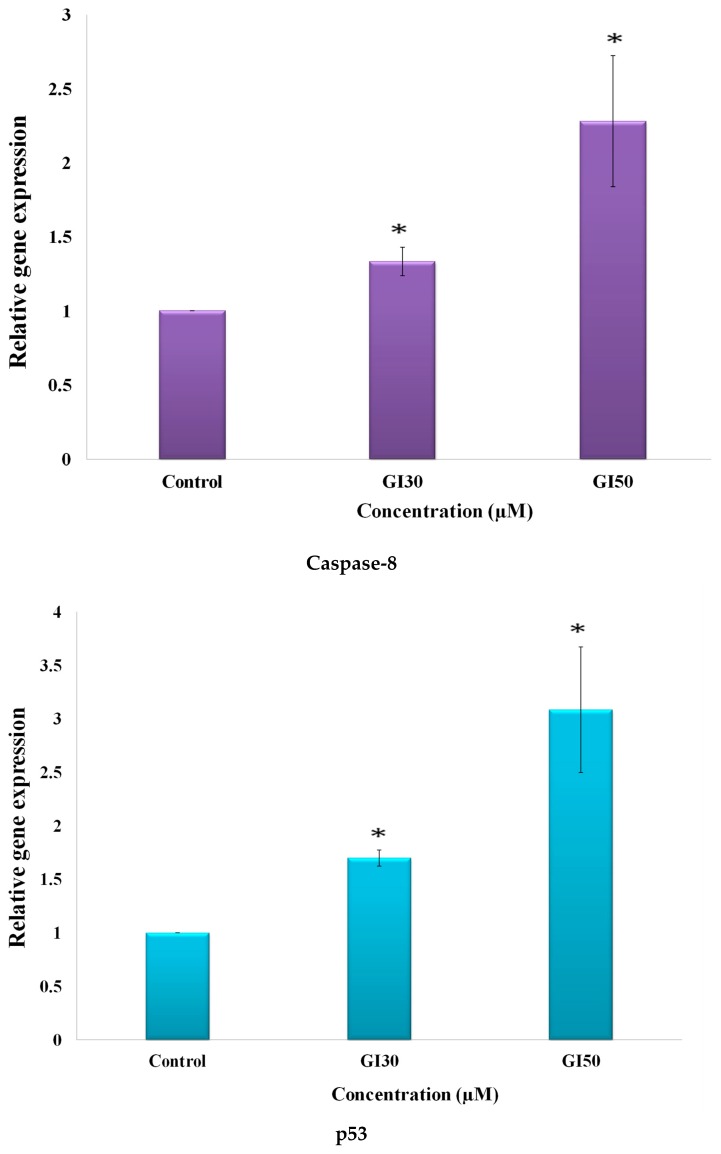
Effect of Epiafzelechin from *C. fistula* on the gene expression for CDK2 (cyclin-dependent kinases-2), β-catenin, caspase-8, and p53 genes in MG-63 cells as detected using RT-PCR. Values are expressed as mean ± SE. * indicates statistically significant difference at (*p* ≤ 0.05).

The quantitative RT-PCR analysis showed an increase of 1.5 and 2-fold in the gene expression for caspase-8 gene and p53 gene at the GI_50_ concentration of Epiafzelechin when compared with untreated control while it indicated a decrease of 0.9 and 0.8 fold in the gene expression for β-catenin gene and CDK2 gene at the GI_50_ concentration of Epiafzelechin when compared with the untreated control in MG-63 cancer cells, as shown in Figure 17.

## 4. Discussion 

Chemoprevention using natural plant products and dietary changes has evolved as a promising strategy to combat cancer. A large number of phytochemicals have been demonstrated to have antioxidant/pro-oxidant, pro-apoptotic, anti-metastatic, antiproliferative, anti-angiogenic effects with efficacy in targeting cellular pathways usually altered in cancer cells and with limited toxicity [24,25]. These phytochemicals target several neoplastic events by preventing the damage to DNA, modulating inflammation, and inhibiting tumor cell proliferation, for the reduction of the overall risk of cancer [26]. In the current study, among all the extract/fractions of *C. fistula* leaves, the CaLE fraction and an isolated Epiafzelechin (CFL1) fraction possessed the potent electron-donating capacity and was able to reduce Fe^3+^ to Fe^2+^. The results demonstrated that these fractions showed the highest reducing ability in comparison to the other isolates of *Cassia fistula* L. (Table 2). The ABTS radical cation scavenging assay, demonstrated the CaLE fraction and Epiafzelechin to effectively inhibit the formation of a preformed ABTS radical cation, a blue-green solution in a concentration-dependent manner (Figure 1). The hydrogen and electron-donating ability of CaLE fraction and Epiafzelechin reduced the preformed radical cation to ABTS. The scavenging ability of the CaLE fraction and Epiafzelechin from *C. fistula* was higher against nitrite ions, which are formed upon the reaction of nitric oxide with oxygen (Figure 2). The addition of sulphanilic acid to this reaction causes the nitrite ions to form diazonium salts that readily coupled with N-(1-naphthyl) ethylenediamine to form a pink azo dye. The CaLE fraction possesses necessary phytocompounds for the elimination of radicals, which in turn, decrease the absorbance. Seccon et al. [27] reported a strong free radical scavenging potential of Epiafzelechin protocatechuate isolated from the bark of *Araucaria angustifolia*. The ethyl acetate fraction of the *Crescentia cujete* leaves and stem bark exhibited potent antioxidant properties in the ferric-reducing power and total antioxidant capacity assays. These properties were attributed to the presence of saponins, steroids, tannins, flavonoids, terpenoids, and glycosides in them [28]. Boussahel et al. [29] showed that methanolic and aqueous extracts from *Retama sphaerocarpa* possess potent antioxidant and antiglycation activities, which were attributed to the presence of flavonols (isorhamnetin, apigenin, kaempferol, and quercetin) and isoflavones (daidzein and genistein derivatives) in them. 

^1^H NMR, ^13^C NMR, DEPT-135, and FT-IR spectroscopic techniques characterized ‘CFL1′ as Epiafzelechin (flavan-3-ol) (Figure 3, Figure 4, Figure 5 and Figure 6). Epiafzelechin isolated from the CaLE fraction showed minimum GI_50_ value of 8.73 μM against the MG-63 cancer cell line followed by IMR-32 and PC-3 with the GI_50_ value of 9.15 and 11.89 μM respectively as compared to the other fractions of *C. fistula* (Figure 8 and Figure 9). A study by Liao et al. [30] revealed the antiproliferative activity of kaempferol against a panel of human cancer cell lines including human stomach carcinoma (SGC-7901) cells, human lung carcinoma (A549) cells, human breast carcinoma (MCF-7) cells, and human cervical carcinoma (HeLa) cells. Phytochemicals via their antioxidant activity mitigate oxidative stress effects by preventing ROS-induced DNA damage, enhancing DNA repair machinery, activating cellular antioxidant defense mechanism, and inhibiting aberrant cell proliferation. On the other hand, in the context of cancer treatment, phytochemicals increase oxidant stress in cancer cells by inactivating pro-survival signals, inhibiting the ROS-scavenging system, inducing DNA damage, activating apoptosis-related signals, and inhibiting signaling pathways favoring cancer cell growth [31]. 

The majority of cancer cells exhibit a remarkable characteristic that is, they fail to undergo apoptosis, which in turn provides them with a survival advantage over normal cells [32]. Deregulation or evasion of cells from apoptosis is the major feature of many types of cancer [33]. Apoptosis is considered a genetically programmed cell death process used to eliminate unwanted cells and is important for cell turnover. Epiafzelechin elicited morphological changes such as cell shrinkage, nuclear fragmentation, apoptotic bodies, membrane blebbing, and chromatin condensation, which was observed using confocal and SEM microscopy (Figure 10 and Figure 11). As Epiafzelechin is capable of restoring the apoptotic functions of cellular proteins, it can be exploited as a potent anticancer agent. Elkady et al. [34] investigated the cytotoxic and apoptosis-inducing potential of flavonoid-rich extract of *Zingibar officinale* against Hepatocellular carcinoma HepG2 cell line. The data obtained revealed that the flavonoid treatment showed the presence of morphological features like membrane blebbing, nuclear condensation, cell shrinkage, detachment, and apoptotic body formation in HepG2 cells. It was found that the fraction isolated from aqueous extract of *Stryphnodendron adstringens* (barbatimão) leaf showed morphological alterations like cell shrinkage, rounding-up, nuclear condensation and reduction of cell diameter and length in the treated two human breast cancer cell lines, MDA-MB-435 and MCF-7. The fraction was reported to be rich in gallic acid, (–)-epicatechin-3-O-gallate, and procyanidin dimer B1 [35].

Reactive Oxygen Species (ROS) generation is one of the effective mechanisms, which results in the induction of apoptosis. Epiafzelechin exhibited a 35.7% increase in the intracellular ROS generation as represented by the M2 cell population in the DCFH-DA stained MG-63 cells using a flow cytometer (Figure 12). Choi and coauthors [36] reported the cytotoxic and apoptogenic potential of kaempferol against the colorectal cancer HCT116 cells through the enhancement in the generation of ROS. In a report by Wei and coworkers [37], it was found that xanthohumol, a prenylated flavonoid isolated from *Humulus lupulus* L. exhibited its antiproliferative and apoptotic effects by the overproduction of ROS in gastric cancer AGS cells. The loss of mitochondrial membrane permeability is another important feature for the determination of apoptosis in cancer cells. The disruption of mitochondrial membrane potential (Δ*Ψm*) is accompanied by the release of cytochrome c. Epiafzelechin effectively disrupted the Δ*Ψm* by 62.6% at the GI_50_ concentration and 73.6% at the GI_70_ concentration in MG-63 cells (Figure 13). Lee et al. [38] reported the cytotoxic effects of (–)-Epigallocatechin-3-gallate (EGCG) in human laryngeal epidermoid carcinoma of the larynx Hep2 cells and found that the induction of apoptosis was accompanied by a decrease in the mitochondrial membrane potential with the release of cytochrome c from the mitochondria. Kaempferol inhibited cell growth and induced cell death by causing depolarization in the mitochondrial membrane potential in a dose- and time-dependent manner in the human glioma cells [39].

Epiafzelechin inhibited the growth of MG-63 cancer cells by arresting the cell population at the G_0_/G_1_ phase of the cell cycle. Epiafzelechin showed a 75% accumulation of cells at the G_0_/G_1_ phase at the GI_70_ concentration (Figure 14). Huang et al. [40] reported the interference of quercetin in the development of malignancy and attributed this to its cell cycle arrest at the G_2_/M phase in oral cancer cells, HSC-3, and TW206. Mu et al. [41] reported the cell growth inhibiting, cell cycle arrest, and immunosuppressive potential of kaempferol in mouse T lymphocytes in vitro and was found to arrest cell cycle at S and G_2_/M phases in a dose-dependent manner. In the Annexin-V/PI double staining assay, the treatment of Epiafzelechin increased the apoptotic cell population in a dose-dependent manner in MG-63 cells (Figure 15). The apoptotic effect of the fraction was detected by the externalization of phosphatidylserine (PS) as the induction of apoptosis causes the translocation of PS from the inner leaflet of the plasma membrane to the outer leaflet in the Annexin-V FITC/PI double-staining method. The combination of 5-fluorouracil and the flavonoid oroxylin from the root of a traditional Chinese medicine *Scutellaria baicalensis* Georgi demonstrated an increase in the population of apoptotic cells as analyzed by Annexin V-FITC/PI double staining in HepG2 human hepatocellular carcinoma cells [42]. Chuwa et al. [43] reported the modulation of mitochondrial pathway and death receptor pathway by kaempferol, a flavonoid through the induction of apoptosis in ER-positive endometrial cancer cell lines as detected by Annexin V-FITC/PI double staining.

Akt is a serine-threonine kinase that is generally activated by lipid products of phosphatidylinositol 3-kinase (PI3K) and is known for its ability to inhibit cell death pathways [44]. The activated Akt phosphorylates numerous protein targets that control cell proliferation, survival, and motility. Akt directly inactivates the proteins involved in apoptosis, including Bad, Forkhead, and procaspase-9 [45]. Notably, in the present study, it was found that the treatment of Epiafzelechin significantly downregulated the level of p-Akt in a dose-dependent manner (Figure 16). Qiao et al. [46] reported the myocardial protective effects of Eupatilin, an active flavone from Artemisia plant species towards cardiomyocyte apoptosis by increasing the activation of Akt. Quercetin, a flavonoid, showed its anticancer activity by the induction of apoptosis in primary effusion lymphoma cells by inhibiting the activation of PI3K/AKT/mTOR and also down-regulating the expression of the prosurvival cellular proteins. The antiproliferative and apoptosis-inducing potential of eight flavonoids was reported by Zhang and coworkers [47] in a variety of human cancer cell lines. The active flavonoids induced apoptosis in cancer cells by downregulating the level of phospho-Akt. 

GSK-3β plays a key role in numerous physiological and cellular processes, including glycogen metabolism, protein synthesis, cell fate determination, cell cycle division, and stem cell maintenance [48,49]. Epiafzelechin treatment decreased the activation of p-GSK-3β expression levels for the induction of apoptosis in osteosarcoma MG-63 cells (Figure 16). Oridonin isolated from *Rabdosia rubescens* is reported to inhibit the proliferation and induced apoptosis in colon cancer COLO205 cells by decreasing the phosphorylation of GSK-3β [50]. Epiafzelechin also suppressed the expression level of β-catenin in a dose-dependent manner as detected by RT-PCR (Figure 17). Park and Choi [51] have found that various polyphenolic flavonoid compounds like genistein, isorhamnentin, kaempferol, and baicalein inhibited the activation of GSK-3β and transcriptional activity of β-catenin/Tcf in the human embryonic kidney (HEK) 293 cells. Quercetin was found to inhibit the proliferation and induced apoptosis in human SW480 colon cancer cells by suppressing the expression of transcriptional activity of β-catenin/Tcf as well as the Wnt/β-catenin signaling pathway [52].

Epiafzelechin was observed to promote the activation of caspase-8 expression, as analyzed using RT-PCR (Figure 17). Caspase-8 is an apical caspase that on following death receptor ligation, initiates, and promotes programmed cell death [53]. Gao et al. [54] reported that kaempferol induced extrinsic apoptosis through the death receptors/FADD/Caspase-8 pathway in human ovarian cancer A2780/CP70 cells. Quercetin treatment induced apoptosis in human oral cancer SAS cells by increasing the expression of Fas, Fas-Ligand, and caspase-8 [55]. Epiafzelechin was shown to downregulate anti-apoptotic protein Bcl-xl, of the Bcl-2 family as detected by western blot analysis (Figure 16). Quercetin was reported to upregulate the expression of pro-apoptotic proteins (Bax and Bad) and downregulated the anti-apoptotic proteins, Bcl-2 and Bcl-xl, thereby suppressing cell proliferation and promoting the induction of apoptosis in various cancer cell lines [56,57,58]. Kaempferol (3,5,7,4-tetrahydroxyflavone) induced mitochondrial-dependent apoptosis in acute human leukemia Jurkat T cells by inactivating the Bcl-xl expression [59]. p53, a tumor suppressor protein is a transcription factor that is mainly activated in response to a wide variety of stresses [60]. MG-63 cells treated with Epiafzelechin showed enhancement in the level of p53 (Figure 17). Fu and coworkers [61] reported that flavonoids and tannins from *Smilax china* L. rhizome showed the inhibition of cell proliferation and induction of apoptosis in human lung adenocarcinoma A549 cells by upregulating the p53 and p-p53 proteins expression. Cyclin-dependent kinase-2 (CDK2) is a member of the protein kinase family. Accumulated evidence demonstrated that overexpression of CDK2 leads to abnormal cell-cycle regulation, which is directly related to hyperproliferation in cancer cells [62]. Epiafzelechin inhibited CDK2 in MG-63 cells (Figure 17). Cho and Park [63] attributed the apoptogenic effects of kaempferol in HT-29 human colon cancer cells to the suppression of protein expression of CDK2. Silymarin, a natural flavonoid, showed its potent cancer chemopreventive efficacy against HT-29 Human colon cancer cells with inhibition in CDK2 kinase activity [64].

## 5. Conclusions 

**Figure 18:**
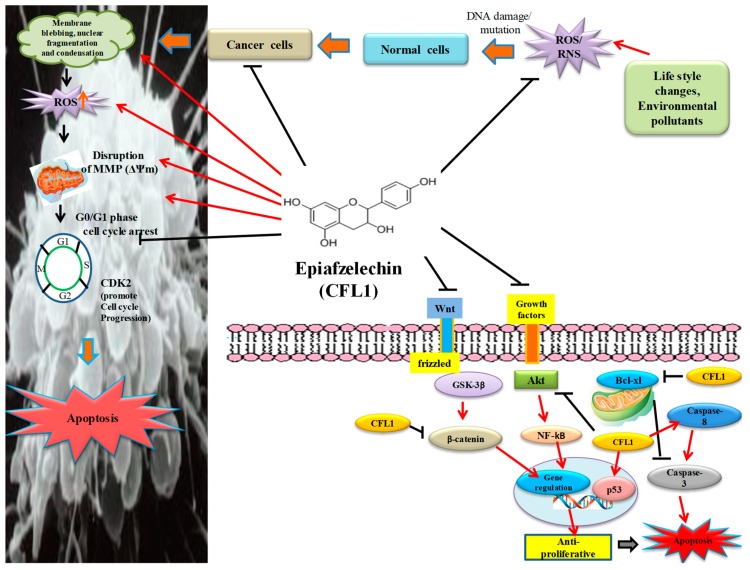
Proposed mechanism of action of the chemopreventive potential of Epiafzelechin (CFL1) isolated from *Cassia fistula* L.

The CaLE fraction with potent antioxidant activity yielded Epiafzelechin, which showed strong antiproliferative effects and induced apoptosis in MG-63 cells as indicated by morphological changes, increased ROS level, decreased MMP level, cell cycle arrest at G_0_/G_1_ phase. Epiafzelechin was found to downregulate the expression of p-Akt, p-GSK-3β, and anti-apoptotic protein, Bcl-xl. Expression of caspase-8 and p53 genes was observed to increase, whereas it decreased the expression of β-catenin and CDK2 genes in MG-63 cells. The above study points towards the remarkable potential of Epiafzelechin in targeting the aberrant signalling pathways of MG63 cells resulting in induction of the apoptosis, as summarized in Figure 18. 

## Figures and Tables

**Table 1:** Primers for cell signaling pathways gene expression quantification.

Primers	Order	Sequence	Accession No.	Product Size	Source
**Caspase-8**	Forward Primer	CGCAAAGGAAGCAAGAACCC	NM_001228.4	192	NCBI
	Reverse Primer	TGCCTGGTGTCTGAAGTTCC			
**β-catenin**	Forward Primer	TGGAAGGTCTCCTTGGGACT	NM_001904.3	200	NCBI
	Reverse Primer	GGCAGGCTCAGTGATGTCTT			
**p53**	Forward Primer	TCACTGAAGACCCAGGTCCA	NM_000546.5	199	NCBI
	Reverse Primer	TTGGCTGTCCCAGAATGCAA			
**CDK2**	Forward Primer	GGCCCTATTCCCTGGAGATTC	NM_001798	180	NCBI
	Reverse Primer	CGTCCATCTTCATCCAGGGG			
**β-actin**	Forward Primer	GTCCTCTCCCAAGTCACACA	T25383	166	NCBI
	Reverse Primer	GCTCATACATCTCAAGTTGGGAC			

**Table 2:** Antioxidant activity of extract/fractions of *C. fistula* leaves in a ferric ion-reducing antioxidant power (FRAP) assay.

Concentration (μg/mL)	Absorbance at 700 nm (Mean ± SE)							
	Ascorbic Acid	CaLM	CaLH	CaLC	CaLE	CaLB	CaLA	CFL1
50	0.207 ± 0.013	0.165 ± 0.002	0.03 ± 0.002	0.164 ± 0.002	0.136 ± 0.04	0.146 ± 0.024	0.061 ± 0.001	0.119 ± 0.003
100	0.456 ± 0.015	0.260 ± 0.007	0.053 ± 0.002	0.221 ± 0.005	0.287 ± 0.004	0.181 ± 0.038	0.096 ± 0.003	0.254 ± 0.057
200	0.643 ± 0.18	0.413 ± 0.018	0.114 ± 0.001	0.342 ± 0.017	0.381 ± 0.022	0.194 ± 0.027	0.164 ± 0.008	0.388 ± 0.025
400	0.814 ± 0.11	0.734 ± 0.008	0.210 ± 0.003	0.588 ± 0.009	0.659 ± 0.022	0.332 ± 0.007	0.287 ± 0.008	0.524 ± 0.026
600	0.875 ± 0.18	0.769 ± 0.048	0.300 ± 0.002	0.677 ± 0.034	0.714 ± 0.033	0.457 ± 0.011	0.362 ± 0.034	0.790 ± 0.012
800	0.985 ± 0.09	0.809 ± 0.026	0.370 ± 0.014	0.723 ± 0.042	0.855 ± 0.013	0.583 ± 0.006	0.490 ± 0.014	0.887 ± 0.033
F-ratio_(12,6)_	832.2 *	135.9 *	492 *	102.8 *	645.3 *	59.65 *	113.4 *	91.08 *
HSD	0.201	0.114	0.029	0.113	0.134	0.107	0.074	0.149

*indicates the significant difference at *p* ≤ 0.05; SE = Standard error.
